# A study on obese patients’ participation in cancer screening programs: an example from Turkey

**DOI:** 10.1590/1806-9282.20240261

**Published:** 2024-08-16

**Authors:** Sezgin Türkoğlu, Mehmet Özen, Remziye Nur Eke, Aysima Bulca Acar

**Affiliations:** 1 Adana Provincial Health Directorate - Adana, Turkey.; 2 University of Health Sciences, Antalya Training and Research Hospital, The Clinic of Family Medicine - Antalya, Turkey.

**Keywords:** Cancer screening, Obesity, Patient participation, Prevention

## Abstract

**OBJECTIVE:**

Obesity is associated with many types of cancers. Despite this, the participation of obese individuals in cancer screenings is limited. The aim of this study was to evaluate the cancer screening-related attitudes of obese patients.

**METHODS:**

The study included 185 obese patients who presented to the obesity center (OC) and 191 obese patients who presented to the family medicine outpatient clinic from October to December 2019. The participants in both groups were first asked whether or not they had ever undergone any cancer screening tests and then provided with relevant training. After 3 months, the participants were contacted again and their attitudes toward cancer screening tests were re-evaluated.

**RESULTS:**

Patients who followed in the OC were found to have higher awareness of and compliance with cancer screening tests than the obese patients admitted to the outpatient clinic. The factors of being female, being followed in the OC, and residing in an urban area were positively associated with participation in cancer screening tests.

**CONCLUSION:**

Monitoring obese patients in target-oriented facilities such as an OC increases the chance of success in the fight against obesity and related health problems.

## INTRODUCTION

Today, obesity is one of the leading causes of preventable death. According to the World Health Organization, in 2016, more than 1.9 billion adults were overweight, of which over 650 million were obese^1^.

In addition to affecting disease-related mortality, obesity has been associated with many diseases^2,3^. Studies also have found that obesity is a risk factor for many cancer types and the rate of cancer attributable to obesity in the world constituted 3.6% of all cancer cases^4,5^. Despite the fact that obesity and cancer are related, research shows that obese people's attitudes toward cancer screening tests appear to be limited and a significant negative correlation has been reported to exist between obesity and participation in cancer screening programs^6-8^.

In the literature, studies investigating the tendency of participation of obese patients in cancer screening tests remain very limited, and likewise, those investigating the participation of such patients followed up in a private center are very few. Therefore, the aim of this study was both to assess the cancer screening status of obese people and to evaluate the possible differences in the attitudes toward cancer screening tests between obese individuals followed up by private centers and those who were not followed up in any center.

## METHODS

### Study design and participants

This is a observational and prospective study and was conducted in a single center. The research population consisted of people who presented to the Family Medicine Outpatient Clinic of the Health Sciences University, Antalya Training and Research Hospital (HSUATRH) between March and May 2020, and those who were followed up in the Health Sciences University Antalya Training and Research Hospital Obesity Center (OC) between October and December 2019. This study included patients having a BMI ≥30 kg/m^2^, being at the age of 18 years and over, having no previous diagnosis of malignancy, not being pregnant or in the postpartum period, and agreeing to participate in the research for both groups.

While determining the number of those to be included in the study in the outpatient group, the fact was that the prevalence of obesity in adults in Turkey was reported to be approximately 30%^9^. In this regard, the sample size was calculated as 244 people (the confidence interval was 95%, and the sampling error was 5% EpiInfo™, 7.2.0.1; sample size and power). However, as the data collection period in the outpatient clinic group overlapped with the COVID-19 pandemic, when many restrictions were imposed on patient admission to outpatient clinics, the data collection process was terminated when 191 participants were included in the study sample.

The study included all of the patients who were followed up at the OC from October to December 2019, and no sampling was done. The number of patients followed at the OC until the end of the study was 198. Because 13 patients had previous diagnoses of malignancy, 185 patients were included in the OC group.

After this process, the cancer screening status of the individuals in the two groups was compared and investigated whether or not the patients followed in the OC developed positive health-related behavior in terms of cancer screening tests.

#### Health Sciences University Antalya Training and Research Hospital Obesity Center

The Center started its operations on November 1, 2018, within the HSUATRH. In the OC, weight loss is targeted to achieve a state of health in those with obesity, and thus, such individuals are informed about health problems caused by obesity through various educational activities: one of these is the relationship between obesity and cancer and the importance of cancer screening.

### Measurement tools

#### Sociodemographic data form

After receiving the written consent of the participants, the researcher asked the research questions through face-to-face interviews with the participants.

The height and weight of the participants were measured by the researchers and their body mass indexes were calculated. The cancer screening programs in Turkey include those for breast, cervical, and colon cancer types, regarding which the participants were questioned.

In our questionnaire, patients were asked questions about their demographic data and whether or not they ever had cancer screening tests done in conformity with age and gender, upon which the patients’ responses were noted. Afterward, they were informed about obesity-related cancer risks in detail, provided with appropriate guidance for any possible missing cancer screening tests and given the prepared information brochure as a print-out. At least 3 months later, the patients were contacted by phone and asked whether or not they got the screenings done as included in the national cancer screening program, upon which their answers were recorded in the research form.

### Ethical approval

Approval for this study was obtained from the Clinical Research Ethics Committee of the UHSATRH with decision number 21/7 (dated 26.09.2019). The study was conducted in accordance with the Declaration of Helsinki.

## RESULTS

Table 1 presents the detailed demographic characteristics of the patients included in the study comparatively.

**Table 1:** Demographic characteristics of the patients.

Demographic characteristics		All patients (n=376)	Patients followed at the OC (n=185)	Outpatients (n=191)	P
Age		50.33±10.89 (19-79)	52.23±9.82 (21-73)	48.49±11.57 (19-79)	**0.001**
Gender	Female	308 (81.9)	174 (94.1)	134 (70.2)	**<0.001**
	Male	68 (18.1)	11 (5.9)	57 (29.8)	
Marital status	Single	40 (10.6)	13 (7)	27 (14.1)	0.082
	Married	291 (77.4)	149 (80.5)	142 (74.3)	
	Separated/divorced	45 (12)	23 (12.4)	22 (11.5)	
Educational background	Illiterate	8 (2.1)	1 (0.5)	7 (3.7)	0.369
	Primary school	168 (44.7)	85 (45.9)	83 (43.5)	
	Secondary school	40 (10.6)	18 (9.7)	22 (11.5)	
	High school	91 (24.2)	46 (24.9)	45 (23.6)	
	University	61 (16.2)	32 (17.3)	29 (15.2)	
	Master's degree/PhD	8 (2.1)	3 (1.6)	5 (2.6)	
Occupation	Unemployed	18 (4.8)	10 (5.4)^a^	8 (4.2)^a^	**<0.001**
	Housewife	181 (48.1)	105 (56.8)^a^	76 (39.8)^b^	
	Worker	81 (21.5)	14 (7.6)^a^	67 (35.1)^b^	
	Civil servant	26 (6.9)	7 (3.8)^a^	19 (9.9)^b^	
	Retired	70 (18.6)	49 (26.5)^a^	21 (11)^b^	
Place of residence	Urban area	344 (91.5)	172 (93)	172 (90.1)	0.310
	Rural area	32 (8.5)	13 (7)	19 (9.9)	
With social security		354 (94.1)	171 (92.4)	183 (95.8)	0.163

OC: obesity center. Results are shown as mean±SD (min-max) or n (%) values. Student's t-test, Pearson chi-square test, and Fisher's exact test. Different lowercase letters in a row indicate statistically significant differences between groups. Bold: Of all the 376 patients included in the study, 81.9% were female and 18.1% were male, with a mean age of 50.33±10.89 years (min.: 19; max.: 79). The ratio of female patients (p<0.001) and mean age (p=0.001) were found to be higher in patients followed in the OC compared with those followed in the outpatient clinic. The housewife and retired rates in OC (p<0.001) and the worker and civil servant rates in the outpatient clinic (p<0.001) were found to be statistically higher.

Table 2 presents the findings regarding the availability of the patients’ cancer screening tests before training.

**Table 2:** The status of patients undergoing cancer screening test prior to the training.

Screening tests*	All patients	Patients followed at the OC	Outpatients	P
Receiving cancer screening (n=376)	254 (67.6)	144 (77.8)	110 (57.6)	**<0.001**
PAP smear/HPV DNA tests (n=308)	158 (51.3)	92 (52.9)	66 (49.3)	0.529
Breast self-exam (n=308)	160 (51.9)	89 (51.1)	71 (53)	0.749
Mammography (n=308)	122 (39.6)	79 (45.4)	43 (32.1)	**0.018**
Colonoscopy/sigmoidoscopy (n=376)	44 (11.7)	23 (12.4)	21 (11)	0.665
Fecal occult blood test (n=376)	42 (11.2)	19 (10.3)	23 (12)	0.586

* The number of patients (n) who needed to have screening tests was specified according to the indication by gender. OC: obesity center. Findings are shown with n (%) values. Pearson chi-square test and Fisher's exact test. Bold: The rate of having cancer screening tests (p<0.001) as well as mammography (p=0.018) and PSA test (p=0.004) prior to the training in patients followed in the OC was found to be statistically significantly higher than that of the outpatients.

Table 3 presents the findings regarding the status of patients undergoing cancer screening tests after the training.

**Table 3:** The status of patients undergoing cancer screening after the training.

Types of screening tests*	All patients	Patients followed in the OC	Outpatients	p
Cancer screening (n=376)	241 (64.1)	146 (78.9)	95 (49.7)	**<0.001**
PAP smear/HPV DNA tests (n=308)	12 (3.9)	8 (4.6)	4 (3)	0.468
Breast self-exam (n=308)	233 (75.6)	144 (82.8)	89 (66.4)	**0.001**
Mammography (n=308)	14 (4.5)	10 (5.7)	4 (3)	0.249
Colonoscopy/sigmoidoscopy (n=376)	2 (0.5)	1 (0.5)	1 (0.5)	0.999
Fecal occult blood test (n=376)	4 (1.1)	2 (1.1)	2 (1)	0.999

* The number of patients (n) who needed to have screening tests was specified according to the indication by gender. OC: obesity center. Findings are shown with n (%) values. Pearson chi-square test and Fisher's exact test. Bold: The rate of having cancer screening done (p<0.001) and breast self-exam (p=0.001) after the training in patients followed up in the OC was found to be statistically higher than those of outpatients.

The rate of patients who presented to the family medicine clinic with no cancer screening at all or who only had it before the training was higher when compared with those who followed in the OC. Likewise, those who had cancer screening both before and after the training among the OC patients (p<0.001) was higher as a rate. While no significant change was observed in the rates of cancer screening tests before and after the training in the OC patients (p=0.652), 33 (30%) of the outpatients who got screening tests done prior to the training were found not to have received any of the relevant screenings after the training (p=0.049).

As a result of the multivariate logistic regression analysis performed to determine the factors that independently affect the status of getting at least one cancer screening done after training in patients who were supposed to get them done, it was found that being a woman (OR: 68.697; 95%CI: 9.01-523.761; p<0.001), being followed at the OC (OR: 2.353; 95%CI: 1.248-4.437; p=0.008), and living in an urban area (OR: 2.507; 95%CI: 1.019-6.166; p=0.045) positively affected the cancer screening status after the training.

## DISCUSSION

This study evaluated a total of 376 patients, including 185 obese patients followed in the OC and 191 obese patients admitted to the outpatient clinic. The obese patients followed in the OC were found to have higher awareness of and compliance with cancer screening tests after the training than the obese patients admitted to the outpatient clinic. Our study revealed that the factors of being female, being followed in the OC, and residing in an urban area were positively associated with participation in cancer screening tests.

In Turkey, the prevalence of obesity in women is approximately twice that of men^10^. Our study revealed that the female gender ratio was statistically significantly higher among the patients followed in the OC compared with those who presented to the outpatient clinic. We consider that this situation was rooted in the fact that the majority of those who presented to the OC were women because the prevalence of obesity was higher in women. Yildirim and Eryilmaz evaluated the profile of patients who presented to the OC in their study conducted in Konya/Turkey and stated that 91.3% of the patients were female and 8.7% were male^11^. These rates seem to be compatible with our study.

In this study, 67.6% of the patients stated that they had at least one cancer screening test done prior to the training. This rate was found to be 77.8% in the patients followed in the OC, whereas it was 57.6% in the patients admitted to the outpatient clinic. The rate of getting cancer screening tests done after receiving relevant training was found to be 64.1% in all patients, 78.9% in those followed in the OC, while it was 49.7% in patients admitted to the outpatient clinic, indicating a significant difference between the two groups. This significant difference between the patients followed in the OC and those admitted to the outpatient clinic in terms of the tendency of getting cancer screening tests done may result from the fact that the health-seeking behavior of the patients followed up in the OC is more advanced, and trainings on "obesity and cancer" and "cancer screenings" are given as a standard procedure in the third module in the OCs^12^.

It is stated that obesity most likely is a barrier to screening for breast and cervical cancers, particularly among white women^13^. The detailed analysis of the cancer screenings shows us that the percentage of women who were supposed to do breast self-exam but never did so was 18.6% in total. This rate was 13.8% for women followed in the OC, while it was 24.8% for those admitted to the outpatient clinic, indicating a significant difference between the two groups. In addition, those who did breast self-exam after training were significantly higher in the patients followed in the OC. In our study, the rate of having mammography in patients who needed that was found to be 49% in total. Bussiere et al. examined the impact of obesity on patients’ access to breast cancer screening tests. That study included women between the ages of 40 and 75 years and reported that the rate of mammography in the last 2 years was 63.3% in obese women and 69.8% in non-obese women^6^. This result supports that obesity is a barrier to breast cancer screening. In obese individuals, reasons such as low self-esteem and poor body perception, as well as fear of humiliation, concern about lack of respect from healthcare providers, and unwillingness to hear weight loss advice may have exerted a negative impact on the desire to seek healthcare.

Our study found that 58% of the patients who were supposed to have a Pap smear screening test actually had it. Also, the rate of those who had a Pap smear screening test after training was higher in the patients followed up in the OC in comparison with those admitted to the outpatient clinic, yet this difference was not statistically significant.

According to the study conducted by Bussiere et al. in France, the rate of having a Pap smear in the last 3 years was found to be 61% in obese patients and 74% in non-obese patients^6^. Richard et al. examined the impact of lifestyle and health-related factors on participation in cervical cancer screening in a population-based study of Swiss women. The study that was conducted with 7,319 women aged 20-69 years revealed that the rate of receiving cervical cancer screening tests in the last 3 years was 72.9%. When multiple variables were adjusted, the odds ratio of participation in cervical cancer screening tests in obese women was found to be significantly lower (OR=0.64; 95%CI)^14^. This study is a good example of the negative impact of obesity on cancer screening participation.

It is known that overweight and obesity are associated with an increased risk of colorectal cancer^15^. In this study that included obese patients, we found the rate of those who got the FOB test done was 16.1% among those who needed to get it done. We also revealed that the rate of those who underwent colonoscopy/sigmoidoscopy in the last 10 years among those who needed that was only 15.4%. A recent study states that colorectal cancer screening starting at 45 or 40 years of age instead of at 50 years of age, or shortening screening intervals, in women and men with obesity appears cost-effective^16^. For this purpose, it can be said that developing special approaches for obese individuals in cancer screening programs can produce cost-effective results.

In this study, the rates of undergoing cancer screening tests and mammography before the training were found to be statistically higher in patients followed up in the OC compared to those of the outpatients, a situation that may be attributed to the positive effect of being followed up in a center on health awareness in general or to the participation of obese individuals with higher health awareness in the OC.

Our study also has some limitations. First of all, the patients followed in the OC were predominantly female, and those who were admitted to the outpatient clinic were different from those in the OC in terms of gender ratios. Second, due to the COVID-19 pandemic, which started in December 2019 with the first case seen in our country in March 2020, our patients’ behavior patterns were affected and their interactions with the outside world decreased accordingly, which may have influenced our patients’ behaviors to participate in cancer screening programs.

## CONCLUSION AND RECOMMENDATIONS

Monitoring obese patients in target-oriented facilities such as an OC increases the chance of success in the fight against obesity and related health problems. In this regard, OCs that prove beneficial in the fight against obesity should be expanded across our country and the world with increased service capacity. We hope that the findings of our study will benefit future research to be conducted in a more comprehensive manner as a multi-center study.
